# Inhibitory Effects of *Chrysanthemum boreale* Essential Oil on Biofilm Formation and Virulence Factor Expression of *Streptococcus mutans*

**DOI:** 10.1155/2015/616309

**Published:** 2015-02-11

**Authors:** Beom-Su Kim, Sun-Ju Park, Myung-Kon Kim, Young-Hoi Kim, Sang-Bong Lee, Kwang-Hee Lee, Na-Young Choi, Young-Rae Lee, Young-Eun Lee, Yong-Ouk You

**Affiliations:** ^1^Wonkwang Bone Regeneration Research Institute, Wonkwang University, Iksan 570-749, Republic of Korea; ^2^Department of Oral Biochemistry, School of Dentistry, Wonkwang University, Iksan 570-749, Republic of Korea; ^3^Department of Food Science & Technology, College of Agriculture & Life Sciences, Chonbuk National University, Jeonju 561-756, Republic of Korea; ^4^Department of Pediatric Dentistry, School of Dentistry, Wonkwang University, Iksan 570-749, Republic of Korea; ^5^College of Education, Wonkwang University, Iksan 570-749, Republic of Korea; ^6^Department of Food and Nutrition, Wonkwang University, Iksan 570-749, Republic of Korea; ^7^Wonkwang Research Institute for Food Industry, Iksan 570-749, Republic of Korea

## Abstract

The aim of the study was to evaluate the antibacterial activity of essential oil extracted from *Chrysanthemum boreale* (*C. boreale*) on *Streptococcus mutans* (*S. mutans*). To investigate anticariogenic properties, and bacterial growth, acid production, biofilm formation, bacterial adherence of *S. mutans* were evaluated. Then gene expression of several virulence factors was also evaluated. *C. boreale* essential oil exhibited significant inhibition of bacterial growth, adherence capacity, and acid production of *S. mutans* at concentrations 0.1–0.5 mg/mL and 0.25–0.5 mg/mL, respectively. The safranin staining and scanning electron microscopy results showed that the biofilm formation was also inhibited. The result of live/dead staining showed the bactericidal effect. Furthermore, real-time PCR analysis showed that the gene expression of some virulence factors such as *gtf*B, *gtf*C, *gtf*D, *gbp*B, *spa*P, *brp*A, *rel*A, and *vic*R of *S. mutans* was significantly decreased in a dose dependent manner. In GC and GC-MS analysis, seventy-two compounds were identified in the oil, representing 85.42% of the total oil. The major components were camphor (20.89%), *β*-caryophyllene (5.71%), *α*-thujone (5.46%), piperitone (5.27%), *epi*-sesquiphellandrene (5.16%), *α*-pinene (4.97%), 1,8-cineole (4.52%), *β*-pinene (4.45%), and camphene (4.19%). These results suggest that *C. boreale* essential oil may inhibit growth, adhesion, acid tolerance, and biofilm formation of *S. mutans* through the partial inhibition of several of these virulence factors.

## 1. Introduction

Dental caries, known as tooth decay or a cavity, is a plaque-related disease of teeth and slowly progressive infectious disease in the dental area [1, 2]. The dental caries disease is caused by specific types of acid-producing bacteria that cause demineralization and destruction of the teeth [3].

*S. mutans* are generally regarded as one of the primary pathogenic bacteria in dental caries [4]. The* S. mutans* adhere to the colonizer and accumulate on the tooth enamel surface by generation of extracellular polysaccharide from fermentable carbohydrates such as sucrose, by action of glucosyltransferases (GTFase) [1, 5]. The carbohydrate metabolism promotes bacteria aggregation to the tooth surface and acid production [1]. The produced acids initiate dissolution of the enamel surface of teeth subsequently leading to localized decalcification [6].

Therefore, inhibition of the growth and biofilm formation of the* S. mutans* is one of the strategies for prevention of dental caries. Although several antiplaque agents have been used, the attempt to search for an effective agent still continued [7, 8]. For example, some studies reported that several natural products derived herb, such as* Mentha longifolia* L.,* Aralia continentalis*, and* Curcuma longa L*, showed the inhibitory effect of dental plaque [9–11].

*C. boreale* is a perennial herb with yellow flowers and belongs to the Asteraceae family. It is widely distributed in wild fields and mountains of East Asia. It is also usually have been used as tea or wine in Korea. The* Chrysanthemum* species herb has been reported as having potential medicinal properties including anti-inflammatory, antiviral, and antibacterial [12–14]. In previous study [15], the essential oil was extracted from* C. boreale* and eighty-seven constituents were identified. Furthermore, the essential oil showed antibacterial activity against several bacteria including* S. mutans*. However there is poorly scientific evidence about effect of essential oil from* C. boreale* on* S. mutans* causing dental plaque formation. Therefore, in this study, we examined influence of essential oil of extracted from* C. boreale* on the growth, acid-production, bacterial attachment, and biofilm formation of* S. mutans*. Furthermore, several virulence factors of* S. mutans*, associated with dental plaque and caries formation, were assessed, and the detailed chemical constituents of* C. boreale* essential* oil* were also analyzed by GC and GC-MS.

## 2. Materials and Methods

### 2.1. Plant Material and Essential Oil

*C. boreale* was collected in October, 2013, at the full flowering stage from plants grown wild in Iksan district in Korea and the aerial parts were used to isolate essential oil. The identity was confirmed by Young-Hoi Kim at the College of Environmental & Bioresource Sciences, Chonbuk National University. Voucher specimen (number: 10-24-13) has been deposited at the Herbarium of College of Environmental & Bioresource Sciences, Chonbuk National University. The aerial parts (leaves, stems, and flowers) of* C. boreale* (1 kg) were finely chopped. The chopped plant materials of* C. boreale* were placed in 5 L round-bottom flask and distilled water was added (3 L). Hydrodistillation was carried out in a Clevenger-type apparatus for 3 hours. The yield of the essential oil of* C. boreale* was 0.84%, based on fresh weight of the plant. The essential oil was stored in a deep freezer (−70°C) to minimize the escape of volatile compounds.

### 2.2. Inhibition of Bacterial Growth

*S. mutans* (ATCC 25175) was purchased from the American Type Culture Collection (ATCC; Rockville, MD, USA) and cultured in brain heart infusion (BHI; Difco, Detroit, MI) broth under aerobic condition at 37°C. To determine inhibitory effect of* C. boreale* on bacterial growth,* S. mutans* was cultured at 37°C in 0.95 mL of BHI broth containing 1% glucose and various concentrations of the essential oil of* C. boreale*. These tubes were inoculated with 0.05 mL of an overnight culture grown in BHI broth (final: 5 × 10^5^ colony-forming units (CFU)/mL), and incubated for 24 h. Also, 0.1% of sodium fluoride (NaF) was used as a positive control. The optical density (OD) of cells was measured at 550 nm using a spectrophotometer. Three replicates were made for each concentration of the test extracts.

### 2.3. Acid Production

Acid production by* S. mutans* was examined to evaluate the effect of the essential oil of* C. boreale*, as described by a previous study [16]. Briefly, the* C. boreale* essential oil was filtered to sterilization using membrane filter with 0.2 *μ*m pore size and added to 0.95 mL of the phenol red broth containing 1% glucose, which was then inoculated with 0.05 mL of the seed culture of* S. mutans*. After 24 h of cultivation, the pH was directly determined in the bacterial growth media using a pH meter (Corning Inc, Corning, NY, USA). The initial pH of BHI with various concentrations of* C. boreale* essential oil was also determined before inoculation of* S. mutans*. Each concentration of the extract was tested in triplicate.

### 2.4. Bacterial Adherence

The effect of* C. boreale* essential oil on bacterial adherence was determined using hydroxyapatite beads (diameter of 80 *μ*m; Bio-Rad, Hercules, CA, USA) in a previously described method [17]. Briefly, hydroxyapatite beads were coated with clarified human saliva and the saliva-coated hydroxyapatite beads (S-HAs) were immersed in bacterial suspension (1 × 10^7^ CFU/mL) with various concentrations of* C. boreale* essential oil. To allow bacteria to be adherent, the mixture was gently agitated for 90 min at 37°C. Following this, S-HAs was rinsed to remove nonadherent bacteria and was transferred to a new tube that contained potassium phosphate buffer. The adherent* S. mutans* onto the S-HAs were dispersed using a sonicator (Fisher Scientific, Springfield, NJ, USA) at 50 W for 30 sec and the supernatants were spread on bacitracin (3.2 mg/mL) contained MSA plate. After 48 h of cultivation, the numbers of colonies were counted.

### 2.5. Biofilm Formation Assay

Biofilm formation was measured by staining with safranin [18] and observation was done by scanning electron microscopy (SEM). Briefly, various concentrations of* C. boreale* essential oil were added to 0.1% sucrose containing BHI broth in 35 mm polystyrene dish or 24-well plate that contained resin teeth (Endura, Shofu Inc., Kyoto, Japan). Then, the culture was created in the allotted broths by inoculating them with seed cultures of* S. mutans* (5 × 10^5^ CFU/mL) and incubated for 24 h. After incubation, the supernatants were removed and the culture dish or resin teeth were rinsed with distilled water. Biofilm formation was stained with 0.1% safranin and photographed. In addition, to observe the biofilm formation using a SEM, the biofilms formed polystyrene dishes were rinsed with distilled water, fixed with 2.5% glutaraldehyde solution, and dehydrated in ethanol gradient series. Then, the samples were sputter-coated with gold and observed by SEM (JOM-6360, JEOL, Tokyo, Japan).

### 2.6. Confocal Laser Scanning Microscopy

To determine the bactericidal effect of* C. boreale* essential oil on* S. mutans* live and dead staining were performed. Briefly, approximately 1 × 10^7^ CFU/mL of* S. mutans* was treated with various concentrations of* C. boreale* essential oil for 24 h at 37°C under aerobic conditions. Then the bacteria were washed with PBS and stained with LIVE/DEAD BacLight Bacterial Viability Kit (Molecular Probes, Eugene, OR, USA) according to the manufacture's protocol. After 15 min of staining, the bacteria were observed using a confocal laser scanning microscopy (LSM 510, Zeiss, Germany).

### 2.7. Real-Time Polymerase Chain Reaction (PCR) Analysis

A real-time PCR was performed to evaluate the effect of* C. boreale* essential oil on gene expression of* S. mutans*. The subminimal inhibitory concentration (0.5–0.25 mg/mL) of the essential oil was treated. After 24 h of culture, total RNA was isolated from* S. mutans* using a Trizol reagent (Bibco-BRL) and cDNA was synthesized. The amplification was performed using a StepOnePlus Real-Time PCR system with QPCR SYBR Green Mixes (Applied Bio system, Foster City, CA, USA). 16S rRNA was used as an internal control. The primer pairs were described by previous report [19] and are listed in Table 1.

### 2.8. GC and GC-MS Analysis

GC analysis was performed on Hewlett-Packard (HP) model 6890 series gas chromatograph, with a flame ionization detector (FID), a split ratio of 30 : 1 using two different fused silica capillary columns, Supelcowax 10 (30 m × 0.32 mm, i.d., 0.25 *μ*m film thickness) and SPB-1 (30 m × 0.32 mm, i.d., 0.25 *μ*m film thickness). The temperature of the column was programmed from 50°C to 230°C at 2°C/min and then kept constant at 230°C for 30 min for Supelcowax 10 column and SPB-1 column was programmed from 40°C to 230°C at 2°C/min and then kept constant at 230°C for 20 min. The injector and detector temperatures for both analyses were 250°C, respectively. The gas carrier was nitrogen at a flow rate of 1.50 mL/min for Supelcowax 10 column and nitrogen at a flow rate of 1.20 mL/min for SPB-1 column. Peak areas were measured by electronic integration. The relative amounts of the individual components are based on the peak areas. The GC-MS was carried out on Agilent 7890A GC and Agilent 5975C mass selective detector (MSD) operating in EI mode at 70 eV, fitted a DB-Wax column (30 m × 0.25 mm, i.d., 0.25 *μ*m film thickness) and SPB-1 column (30 m × 0.25 mm, i.d., 0.25 *μ*m film thickness). The temperature of the column were programmed from 40°C to 230°C at 2°C/min and then kept constant at 230°C for 30 min for both analyses. The injector and interface temperatures were 250°C, respectively. The gas carrier was helium at a flow rate of 1.50 mL/min for both analyses. The identification of the chemical constituents was based on comparison of their mass spectral spectra with those of Wiley7n/NIST05 mass spectra libraries, and then the compounds of MS matching similarity ≥ 90% were selected as results. Linear retention indices were calculated for each component with the retention time of* n*-alkane series (C_6_–C_26_) [20] under same GC operating conditions with the sample. They were compared with their retention indices available in the literatures [21, 22] or NIST gas chromatographic retention data webbook (http://webbook.nist.gov/chemistry/) database.

### 2.9. Statistical Analysis

All experiments were performed in triplicate. Data were analyzed using the statistical package for social sciences (SPSS, Chicago, IL, USA). The data were expressed as the mean ± standard deviation (SD) values. The statistical analysis was evaluated by one-way ANOVA. Values of *P* < 0.05 were considered as statistically significant.

## 3. Results

### 3.1. Bacterial Growth Inhibition by* C. boreale*

**Figure 1:**
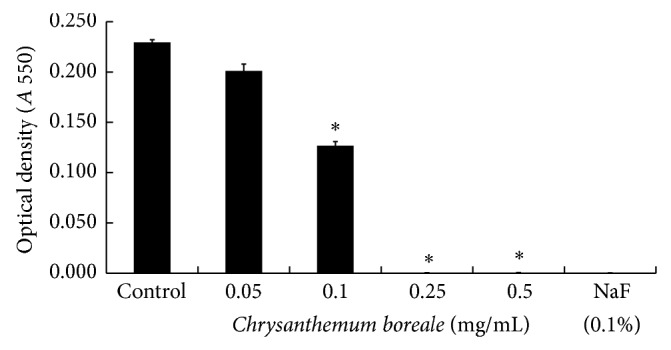
Effect of* Chrysanthemum boreale *(*C. boreale*) essential oil on growth of* Streptococcus mutans *(*S. mutans*)*. S. mutans* was inoculated into BHI broth with various concentrations of* C. boreale* essential oil and incubated for 24 h. Antibacterial activity against* S. mutans* was shown in presence of* C. boreale* essential oil at concentration ranging from 0.1 mg/mL to 0.5 mg/mL. Each value is expressed as a mean ± standard deviation (SD). 0.1% of sodium fluoride (NaF) was used as a positive control. Significance was determined at ^*^ *P* < 0.05 when compared with the control.

In the study, we firstly investigated the antibacterial activity of the essential oil of* C. boreale* against* S. mutans*. The bacteria were treated with 0.05, 0.1, 0.25, and 0.5 mg/mL of* C. boreale* essential oil. When treated with 0.1% NaF, as a positive control, the manifested significant inhibition was shown. When treated with 0.1 mg/mL of the essential oil, the bacterial growth was significantly inhibited. In addition, significant inhibition was shown at concentrations 0.25 mg/mL and 0.5 mg/mL of essential oil in comparison to the control group (Figure 1) (*P* < 0.05).

Furthermore, the manifested significant inhibition was shown at concentrations higher than 0.25 mg/mL and 0.5 mg/mL in comparison to the control group.

### 3.2. Inhibition of Acid Production

To determine whether the* C. boreale* essential oil inhibits the acid production in* S. mutans*, the bacteria were cultured in the presence of various concentrations (0.05–0.5 mg/mL) of the essential oil and the pH change was measured. As shown in Table 2, the pH was significantly decreased at control group (pH 5.47 ± 0.05). However, the pH decrease was significantly inhibited at positive group (0.1% NaF, pH 7.37 ± 0.05). Although the pH decrease was not inhibited at 0.05–0.1 mg/mL of* C. boreale* essential oil, when treated with 0.25 mg/mL and 0.5 mg/mL of* C. boreale* essential oil, the pH decrease was also significantly inhibited and the inhibition levels was similar to the positive group. These results indicate that the* C. boreale* essential oil may inhibit the organic acid production by* S. mutans*.

### 3.3. Inhibitory Effect of* C. boreale* Essential Oil on* S. mutans* Adherence

**Figure 2:**
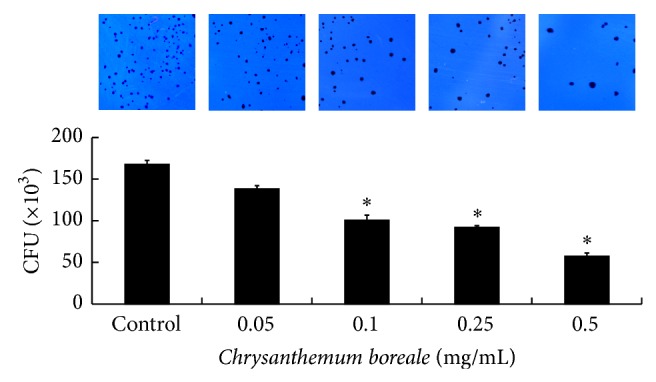
Effect of* Chrysanthemum boreale *(*C. boreale*) essential oil on colony-forming units (CFU) of* Streptococcus mutans *(*S. mutans*)*. S. mutans* was inoculated into BHI broth with various concentrations of* C. boreale* essential oil and incubated for 24 h. The CFU of* S. mutans* that adhered to saliva-coated hydroxyapatite beads that were treated with various concentration of* C. boreale* essential oil are shown. When treated with 0.1–0.5 mg/mL of* C. boreale* essential oil, adherence was significantly repressed. Each value is expressed as a mean ± standard deviation (SD). Significance was determined at ^*^ *P* < 0.05 when compared with the control. 0.1% of sodium fluoride (NaF) was used as a positive control.

We tested the inhibitory effect of* C. boreale* essential oil on the ability of* S. mutans* to adhere to S-HAs. When treated with* C. boreale* essential oil, the* S. mutans* was significantly inhibited in a dose dependent manner. The adherence onto S-HAs particularly was significantly inhibited at concentration of 0.1–0.5 mg/mL of* C. boreale* essential oil (Figure 2).

### 3.4. Bactericidal Effect of* C. boreale* Essential Oil on* S. mutans*

**Figure 3:**
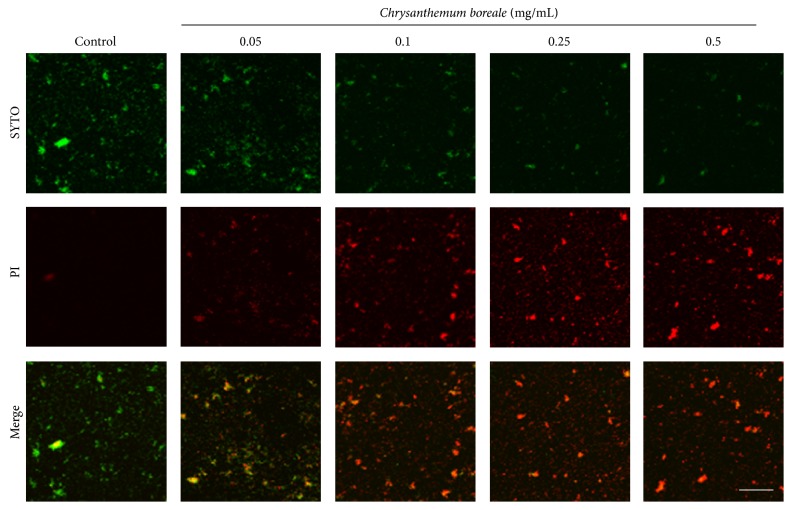
Bactericidal effect of* Chrysanthemum boreale *(*C. boreale*) essential oil. Cultured* Streptococcus mutans *(*S. mutans*) were treated with* C. boreale* essential oil and stained with LIVE/DEAD BacLight Bacterial Viability Kit. Treatment with* C. boreale* essential oil showed bactericidal effect on* S. mutans* in a dose dependent manner. Living bacteria was stained by SYTO 9 as green color and dead bacteria was stained by PI as a red color. Scale Bar = 100 *μ*m.

To evaluate bactericidal effect of* C. boreale* essential oil,* S. mutans* were cultured in presence of various concentrations (0.05–0.5 mg/mL) of the essential oil and stained with LIVE/DEAD BacLight Bacterial Viability Kit and they were observed using confocal laser scanning microscopy. Treatment with* C. boreale* essential oil decreases living bacteria (green fluorescence labeled cell stained by SYTO 9) and increases dead bacteria (red fluorescence labeled cell stained by PI) in a dose dependent manner (Figure 3). This result suggests that* C. boreale* essential oil has bactericidal effect on* S. mutans*.

### 3.5. Inhibitory Effect of* C. boreale* Essential Oil on Biofilm Formation

**Figure 4:**
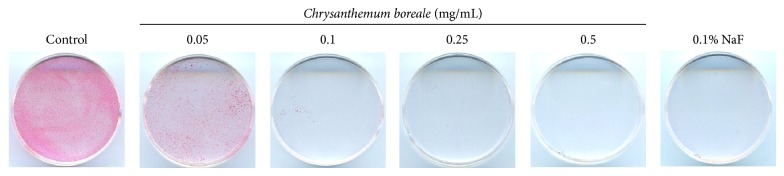
Effect of* Chrysanthemum boreale *(*C. boreale*) essential oil on biofilm formation on polystyrene dishes by* Streptococcus mutans *(*S. mutans*)*. S. mutans* was inoculated into BHI broth with various concentrations of* C. boreale* essential oil and cultured for 48 h. The biofilm that formed on the polystyrene dish surface was measured by staining with 0.1% safranin. Biofilm formation was also significantly inhibited at 0.1 mg/mL and 0.5 mg/mL of the* C. boreale* essential oil. 0.1% of sodium fluoride (NaF) was used as a positive control.

**Figure 5:**
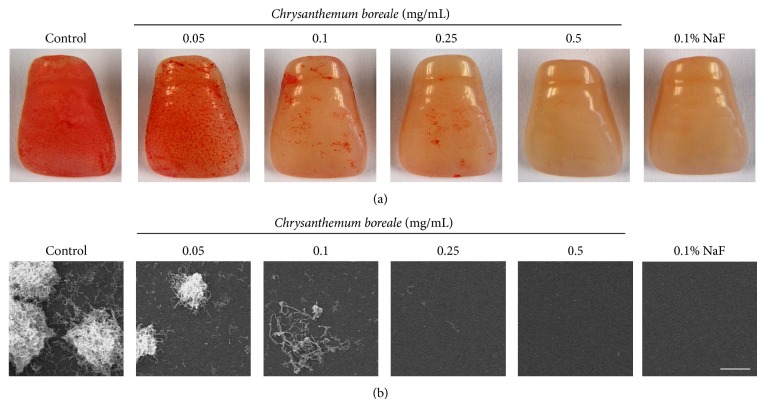
Effect of* Chrysanthemum boreale *(*C. boreale*) essential oil on biofilm formation on resin teeth surface.* Streptococcus mutans *(*S. mutans*) biofilm on resin tooth surface were incubated in various concentration of* C. boreale* essential oil (a). Biofilm formation was significantly inhibited at 0.05–0.25 mg/mL of* C. boreale* essential oil. Also biofilm formation was completely inhibited at 0.5 mg/mL of* C. boreale* essential oil. Scanning electron microscopy image of* S. mutans* biofilm formation on resin tooth surfaces (b). 0.1% of sodium fluoride (NaF) was used as a positive control. Scale bar represents 25 *μ*m.

To determine whether* C. boreale* essential oil inhibits biofilm formation by* S. mutans*, the bacteria had been cultured in the presence of various concentrations of* C. boreale* essential oil in polystyrene dishes. As a result of safranin staining, the biofilm formation by* S. mutans* was significantly inhibited by treatment with* C. boreale* essential oil in a dose dependent manner. When treated with 0.1% NaF (positive control), complete inhibition was shown. In addition, the biofilm formation was also significantly inhibited at 0.1 mg/mL and 0.5 mg/mL of the essential oil (Figure 4). Also, we observed biofilm formation on the surface of resin teeth by safranin staining and SEM observation. Treatment with 0.05 mg/mL of* C. boreale* essential oil slightly inhibited biofilm formation by* S. mutans* and significantly inhibited at concentration 0.1–0.5 mg/mL of* C. boreale* essential oil (Figure 5(a)). Also the SEM image showed consistent result with safranin staining of resin teeth (Figure 5(b)).

### 3.6. Inhibitory Effect of* C. boreale* Essential Oil on Expression of Virulence Factor

**Figure 6:**
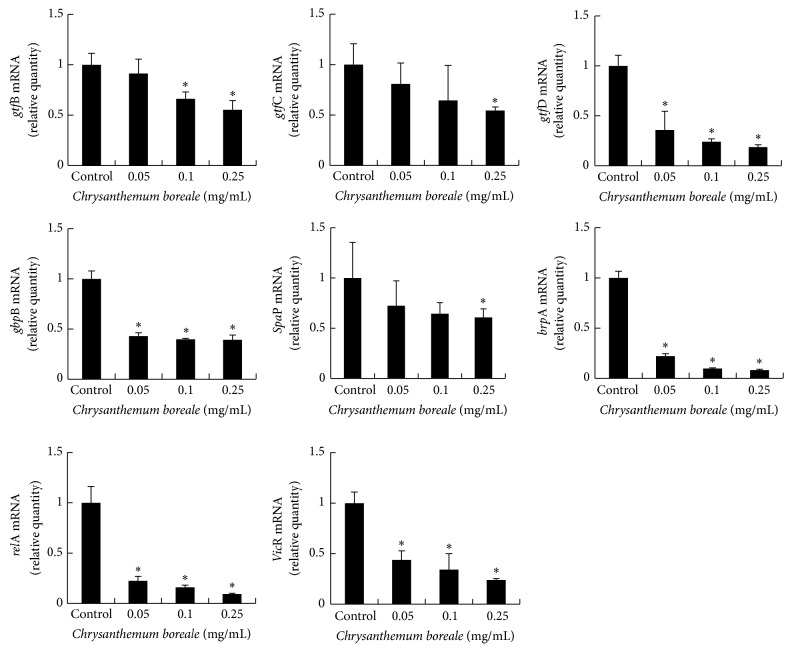
Real-time PCR analysis of mRNA expressions of several virulence factor genes.* Streptococcus mutans *(*S. mutans*) was cultured and treated with various concentrations of* Chrysanthemum boreale *(*C. boreale*) essential oil and real-time PCR analysis was performed as described in the Materials and Methods.* gtf*B,* gtf*C,* and gtf*D were significantly inhibited at 0.1–0.25 mg/mL, 0.25 mg/mL, and 0.05–0.25 mg/mL of* C. boreale* essential oil, respectively. In addition,* gbp*B,* brp*A,* rel*A, and* vic*R expressions were significantly inhibited at 0.05–0.25 mg/mL. The expression of* spa*P was significantly inhibited at 0.25 mg/mL of* C. boreale* essential oil. Each value is expressed as a mean ± standard deviation. Significance was determined at ^*^ *P* < 0.05 when compared with the control.

To assess the effect of* C. boreale* essential oil on the gene expression of virulence factors,* S. mutans* was cultured in presence of 0.05–0.25 mg/mL of* C. boreale* essential oil and the gene expressions of virulence factors were evaluated by real-time PCR (Figure 6). After treatment with* C. boreale* essential oil, firstly genetic expression of* gtf*B,* gtf*C, and* gtf*D, which encode GTFase B, C, and D proteins, respectively, was evaluated. The expression of* gtf*B was significantly decreased when* S. mutans* was treated with 0.1 mg/mL and 0.25 mg of* C. boreale* essential oil and* gtf*C was significantly decreased at 0.25 mg/mL of* C. boreale* essential oil. However, the expression of* gtf*D was significantly decreased by* C. boreale* essential oil at concentration of 0.05–0.25 mg/mL. The expression of* Spa*P and* gbp*B, which contribute to bacterial adherence, was also decreased at 0.25 mg/mL and 0.05–0.25 mg/mL of* C. boreale* essential oil, respectively. The expression of* brp*A and* rel*A, which are related with acid tolerance and* vic*R, which is associated with regulating the expression of* gbp*B,* gtf*B,* gtf*C, and* gtf*D, was also decreased by* C. boreale* essential oil treatment at the concentration of 0.05–0.25 mg/mL.

### 3.7. GC and GC-MS Analysis

The chemical composition of* C. boreale* essential oil identified by GC and GC-MS analysis was shown in Table 3 together with their major constituent. Seventy-two compounds were identified in the oil, representing 85.42% of the total oil. All unidentified compounds were minor components. The major components were camphor (20.89%), *β*-caryophyllene (5.71%), *α*-thujone (5.46%), piperitone (5.27%),* epi*-sesquiphellandrene (5.16%), *α*-pinene (4.97%), 1,8-cineole (4.52%), *β*-pinene (4.45%), and camphene (4.19%) (Table 3).

## 4. Discussion

*C. boreale* are frequently used as a tea or wine in oriental medicine and their medicinal effects such as anti-inflammatory, ant-viral, and antibacterial have been reported [12–14]. Previously, we reported that the* C. boreale* essential oils were extracted and it was identified that the essential oils were composed of eighty-seven constituents where major components were camphor, *α*-thujone, cis-chrysanthenol, 1,8-cineole, *α*-pinnen, and *β*-caryophyllene. Furthermore, the essential oil exhibited the inhibitory effect on growth of several bacteria including* S. mutans* [15]. However, there is no report on its potential effect on the cariogenic properties such as bacterial growth, adherence, biofilm formation, and acid production.

To evaluate anticariogenic properties of* C. boreale* essential oil,* S. mutans* was used because the bacteria is considered as a major bacterium for the formation of dental caries [5, 23]. Our results showed that growth of* S. mutans* was suppressed by treatment with* C. boreale* essential oil. Furthermore, the live/dead staining results also showed that* C. boreale* essential oil has an antibactericidal effect against* S. mutans*. These results suggested that* C. boreale* essential oil has a potential for anticariogenic effect because the inhibition of the growth of* S. mutans *is one of the strategies for prevention of dental caries.

In dental plaque formation, pH is one of the major causes because low pH leads to demineralized tooth enamel and favors the occurrence of the dental caries.* S. mutans* can metabolize dietary sugars and produce organic acid and the produced acids and it is induced by acidic environment in the mouth [24]. Therefore, the alternation of pH is used as an indicator to determine the effect of anticariogenic agents. In this study,* C. boreale* essential oil inhibited the decrease of pH induced by* S. mutans* and the result suggests that* C. boreale* essential oil may be inhibiting dental caries through inhibition of acid production by* S. mutans.*

Furthermore, in the creation of dental plaque process, synthesized extracellular glucan by* S. mutans* is generally regarded as being a major factor [25]. Glucans induce bacterial adherence and result in the formation of dental biofilm [26]. Herein, we examined whether* C. boreale* essential oil can inhibit the ability of* S. mutans* to adhere to S-HAs. Our result showed that* C. boreale* essential oil significantly inhibited bacterial adhesion. In addition, biofilm formation by* S. mutans* was also inhibited by treatment with* C. boreale* essential oil cultured both on polystyrene dishes and on surface of resin teeth. These results suggested that* C. boreale* essential oil directly inhibits the attachment and biofilm formation by* S. mutans*.

Several virulence factors of* S. mutans* are associated with cariogenicity such as bacterial adhesion [27], biofilm formation [28], and acid tolerance [29]. In this study, to evaluate correlation between inhibitory effect by* C. boreale* essential oil and virulence factors expression, we determined the mRNA expression level of several virulence factors such as* gtf*B,* gtf*C,* gtf*D,* gbp*B,* spa*P,* brp*A,* rel*A, and* vic*R, using a real-time PCR analysis. Firstly we evaluated the gene expression level of* gtf*B,* gtf*C, and* gtf*D, which encode the glucosyltransferases (GTFase) B, C, and D. GTFase are recognized as essential virulence factor because these enzymes synthesize glucan from sucrose; the synthesized glucans provide binding site for bacterial adhesion [27]. Besides GTFase virulence factors,* gbp*B, which encodes surface-associated glucan binding protein (GBP), are also a required factor for bacterial adhesion because the protein mediates interaction between cell surface and glucan [30]. Furthermore,* S. mutans* expressed* spa*P gene which encodes* Spa*P protein and the protein contributes adhesion of* S. mutans* [31, 32]. In this study,* C. boreale* essential oil significantly inhibited the transcription level of* gtf*B,* gtf*C,* gtf*D,* gbp*B, and* spa*P. In biofilm formation process by* S. mutans*,* brp*A and* rel*A gene play critical roles.* brp*A is associated with biofilm regulation [28] and* rel*A gene plays a major role in several processes including biofilm formation, glucose uptake, and acid tolerance [33, 34]. Also* vic*R gene is reportedas a regulatory gene of other virulence factors such as* gbp*B,* gtf*B,* gtf*C, and* gtf*D [19]. Based on our results of real-time PCR, the expression of* brp*A,* rel*A, and* vic*R was also repressed when treated with* C. boreale* essential oil. The chemical constituents of* C. boreale* were analyzed with GC and GC-MS. Seventy-two compounds were identified in the oil, representing 85.42% of the total oil. All unidentified compounds were minor components. The major components were camphor (20.89%), *β*-caryophyllene (5.71%), *α*-thujone (5.46%), piperitone (5.27%),* epi*-sesquiphellandrene (5.16%), *α*-pinene (4.97%), 1,8-cineole (4.52%), *β*-pinene (4.45%), and camphene (4.19%). Some previous results reported that the essential oils from* C. coronarium* and* C. indicum* contain monoterpene hydrocarbons and oxygenated monoterpenes such as *α*-pinene, *β*-pinene, camphene, 1,8-cineole, *α*-thujone, camphor, and sesquiterpene hydrocarbon *β*-caryophyllene as major components and these components contribute to antimicrobial and antifungal properties of the oil [35, 36].

## 5. Conclusion

This study has proved that* C. boreale* essential oil exhibited significant inhibition of bacterial growth, adherence capacity, and acid production of* S. mutans*. Furthermore,* C. boreale* essential oil also inhibited the transcription level of several virulence factors such as* gtf*B,* gtf*C,* gtf*D,* gbp*B,* spa*P,* brp*A,* rel*A, and* vic*R of* S. mutans*. In GC and GC-MS analysis, the major components were camphor, *β*-caryophyllene, *α*-thujone, piperitone,* epi*-sesquiphellandrene, *α*-pinene, 1,8-cineole, *β*-pinene, and camphene. Therefore,* C. boreale* essential oil appears to be a promising new agent that may prevent dental caries.

## Figures and Tables

**Table 1:** Oligonucleotide primers that were used in this study.

Gene^*^	Gene description	Primer sequences (5′-3′)	
*gtf*B	Glucosyltransferase-I	Forward: Reverse:	AGCAATGCAGCCAATCTACAAAT ACGAACTTTGCCGTTATTGTCA

*gtf*C	Glucosyltransferase-SI	Forward: Reverse:	GGTTTAACGTCAAAATTAGCTGTATTAGC CTCAACCAACCGCCACTGTT

*gtf*D	Glucosyltransferase-S	Forward: Reverse:	ACAGCAGACAGCAGCCAAGA ACTGGGTTTGCTGCGTTTG

*brp*A	Biofilm regulatory protein A	Forward: Reverse:	GGAGGAGCTGCATCAGGATTC AACTCCAGCACATCCAGCAAG

*spa*P	Cell surface antigen SpaP	Forward: Reverse:	GACTTTGGTAATGGTTATGCATCAA TTTGTATCAGCCGGATCAAGTG

*gbp*B	Secreted antigen GbpB/SagA	Forward: Reverse:	ATGGCGGTTATGGACACGTT TTTGGCCACCTTGAACACCT

*rel*A	GTP pyrophosphokinase	Forward: Reverse:	ACAAAAAGGGTATCGTCCGTACAT AATCACGCTTGGTATTGCTAATTG

*vic*R	Response regulator	Forward: Reverse:	TGACACGATTACAGCCTTTGATG CGTCTAGTTCTGGTAACATTAAGTCCAATA

*16S r*RNA	16S rRNA	Forward: Reverse:	CCTACGGGAGGCAGCAGTAG CAACAGAGCTTTACGATCCGAAA

^*^Based on the NCBI *S. mutans* genome database.

**Table 2:** Effect of essential oil of *C. boreale* on acid production of *S. mutans*.

Conc. (mg/mL)	pH (before incubation)	pH (after incubation)
Control	7.00 ± 0.00	5.47 ± 0.05
0.05	7.00 ± 0.00	5.43 ± 0.05
0.1	7.00 ± 0.00	5.33 ± 0.05
0.25	7.00 ± 0.00	7.33 ± 0.05^*^
0.5	7.00 ± 0.00	7.33 ± 0.05^*^
0.1% NaF	7.00 ± 0.00	7.37 ± 0.05^*^

Data (pH) are represented as mean ± standard deviation. ^*^ *P* < 0.05 when compared with the control group after incubation.

**Table 3:** GC and GC-MS analysis of the essential oil isolated from *C. boreale*.

Peak no.^a^	Components	Retention index		Peak area (%)^d^
		Polar^b^	Apolar^c^	
Monoterpene hydrocarbons				**(19.66)**
1	Tricyclene	1009	920	0.18
2	*α*-Pinene	1027	933	4.97
3	*α*-Thujene	1030	925	0.23
4	Camphene	1070	945	4.19
5	*β*-Pinene	1110	970	4.45
6	Sabinene	1123	966	0.61
10	Myrcene	1167	984	0.92
11	*α*-Terpinene	1181	1007	0.48
13	Limonene	1199	1020	0.65
16	*cis*-*β*-Ocimene	1239	1029	0.87
17	*γ*-Terpinene	1249	1050	0.47
18	*p*-Cymene	1276	1022	1.34
19	Terpinolene	1286	1078	0.30
Oxygenated monoterpenes				**(47.23)**
12	2,3-Dehydro-1,8-cineole	1191	976	0.05
14	1,8-Cineole	1212	1020	4.52
22	*α*-Thujone	1427	1090	5.46
23	*β*-Thujone	1441	1096	1.04
28	Camphor	1518	1124	20.89
30	Linalool	1537	1101	0.10
31	*trans*-Sabinene hydrate	1562	1051	0.40
32	*trans*-Chrysanthenyl acetate	1569	1186	0.46
33	Bornyl acetate	1576	1268	0.69
35	Terpinen-4-ol	1599	1165	0.72
36	Lavandulyl acetate	1608	—	0.08
37	Myrtenal	1621	1167	0.15
38	Umbellulone	1639	1149	0.28
39	Pinocarveol	1651	1124	0.29
40	*p*-Mentha-1,5-dien-8-ol	1662	1169	0.60
43	*α*-Terpineol	1697	1174	0.38
44	Borneol	1701	1146	1.94
48	Piperitone	1725	1224	5.27
49	Carvone	1731	1212	1.14
50	*cis*-Chrysanthenol	1761	1157	2.07
53	Myrtenol	1795	1179	0.32
54	*trans*-Carveol	1833	1181	0.09
55	Geraniol	1852	—	0.07
56	Geranyl acetone	1854	—	0.08
57	*cis*-Carveol	1860	1196	0.14
Sesquiterpene hydrocarbons				**12.51**
25	*α*-Guaiene	1470	—	0.09
27	*α*-Copaene	1493	1370	0.32
29	Berkheyaradulen	1527	1377	0.19
34	*β*-Caryophyllene	1590	1412	5.71
41	*cis*-*β*-Farnesene	1671	—	0.38
42	*β*-Selinene	1676	1488	0.15
45	*epi*-Sesquiphellandrene	1707	—	5.16
46	Widdrene	1710	—	0.09
47	Zingiberene	1714	1496	0.42
51	*ar*-Curcumene	1780	1484	0.28
Oxygenated Sesquiterpenes				**(4.44)**
58	Caryophyllene oxide	1975	1561	2.08
59	Viridiflorol	2042	1569	0.10
60	Nerolidol	2049	1555	0.38
61	Elemol	2072	—	0.40
62	Spathulenol	2118	1563	0.42
64	Torreyol	2167	1606	0.15
67	*epi*-Globulol	2217	1589	0.44
68	Farnesol	2244	1688	0.03
70	*α*-Costol	2301	—	0.29
71	Caryophyllenol II	2349	—	0.07
72	Farnesol (isomer)	2353	1704	0.08
Others				**(1.58)**
5	*n*-Hexanal	1087	835	0.07
8	Butyl benzene	1126	938	0.11
9	2-Methylpropylbenzene	1133	1050	0.14
15	2-Pentyl furan	1236	981	0.04
20	6-Methyl-5-hepten-2-one	1341	—	0.06
21	*n*-Hexanol	1356	882	0.10
24	1-Octen-3-ol	1454	966	0.21
26	2,2,4-Trimethyl-2-cyclohexene carbaldehyde	1474	1040	0.04
52	Methyl salicylate	1789	1169	0.07
63	Eugenol	2164	1327	0.29
65	Thymol	2185	1275	0.34
66	Carvacrol	2212	1275	0.05
69	Decanoic acid	2263	—	0.06

Total identified				(85.42)

^a^Numbering refers to the elution order on Supelcowax 10 column.

^b^Retention index on polar Supelcowax 10 column.

^c^Retention index on apolar SPB-1 column.

^d^Peak area percentage is based on polar Supelcowax 10 column, and values represent averages of three determinations.
